# Long-term follow-up in pediatric intensive care—a narrative review

**DOI:** 10.3389/fped.2024.1430581

**Published:** 2024-07-01

**Authors:** Ashfaque Quadir, Marino Festa, Michelle Gilchrist, Kate Thompson, Natalie Pride, Shreerupa Basu

**Affiliations:** ^1^ Paediatric Intensive Care Unit, The Children's Hospital at Westmead, Sydney, NSW, Australia; ^2^ The University of Sydney, Sydney, NSW, Australia; ^3^ Kids Neuroscience Centre, The Children's Hospital at Westmead, Sydney, NSW, Australia; ^4^ Department of Cardiology, Boston Children's Hospital, Boston, MA, United States

**Keywords:** long-term follow-up, pediatric intensive care, outcomes, post intensive care syndrome, PICS-p

## Abstract

Pediatric intensive care is a rapidly developing medical specialty and with evolving understanding of pediatric pathophysiology and advances in technology, most children in the developed world are now surviving to intensive care and hospital discharge. As mortality rates for children with critical illness continue to improve, increasing PICU survivorship is resulting in significant long-term consequences of intensive care in these vulnerable patients. Although impairments in physical, psychosocial and cognitive function are well documented in the literature and the importance of establishing follow-up programs is acknowledged, no standardized or evidence-based approach to long-term follow-up in the PICU exists. This narrative review explores pediatric post-intensive care syndrome and summarizes the multifactorial deficits and morbidity that can occur in these patients following recovery from critical illness and subsequent discharge from hospital. Current practices around long-term follow-up are explored with discussion focusing on gaps in research and understanding with suggested ways forward and future directions.

## Introduction

Paediatric intensive care has developed rapidly since its advent over 60 years ago and faces unique challenges as a medical specialty. With evolving understanding of paediatric pathophysiology and advances in technology, paediatric intensive care teams are providing ever-improving critical care with a vast majority of children in the developed world now surviving to intensive care and hospital discharge, with a recent systematic review reporting paediatric intensive care mortality rates as low as 1.3% (1). Importantly, these authors simultaneously report that children following PICU admission have significantly increased physical and psychosocial morbidity with associated poorer quality of life. Increased PICU survivorship has led to a greater focus on long-term outcomes and the need for follow-up of survivors of critical care in the paediatric intensive care literature. Many authors describe the need to establish robust and sustainable follow-up programs for these vulnerable children to ensure that they are able to continue to live healthy lives and achieve their full potential (2, 3). With this context in mind, this narrative review explores the why, what and who of long-term follow-up in paediatric intensive care.

## The “Why”: paediatric post-intensive care syndrome

Despite reducing case fatality rates in complex paediatric illnesses due to advances in paediatric intensive care, survival of children beyond hospital discharge is frequently complicated by significant morbidity and impaired quality of life (1, 3, 4). The need to identify and define these outcomes is essential to ensure that an appropriate and considered approach can be taken for longer term follow-up and management. Post-intensive care syndrome (PICS) is a conceptual term that has been used in the literature to describe a range of physical, psychological, cognitive and/or, social impairments that persist in patients that have been discharged from the ICU. Given the complexity and heterogeneity of both patients and illnesses that require intensive care and the variety of often invasive treatment modalities used, the epidemiology and clinical phenotypes within this “syndrome” are yet to be fully defined (5, 6). Defining PICS has had renewed importance following the recent COVID-19 pandemic which has led to a high number of adult intensive care survivors with chronic symptoms and long-term complications following their critical illness (7).

**Figure 1:**
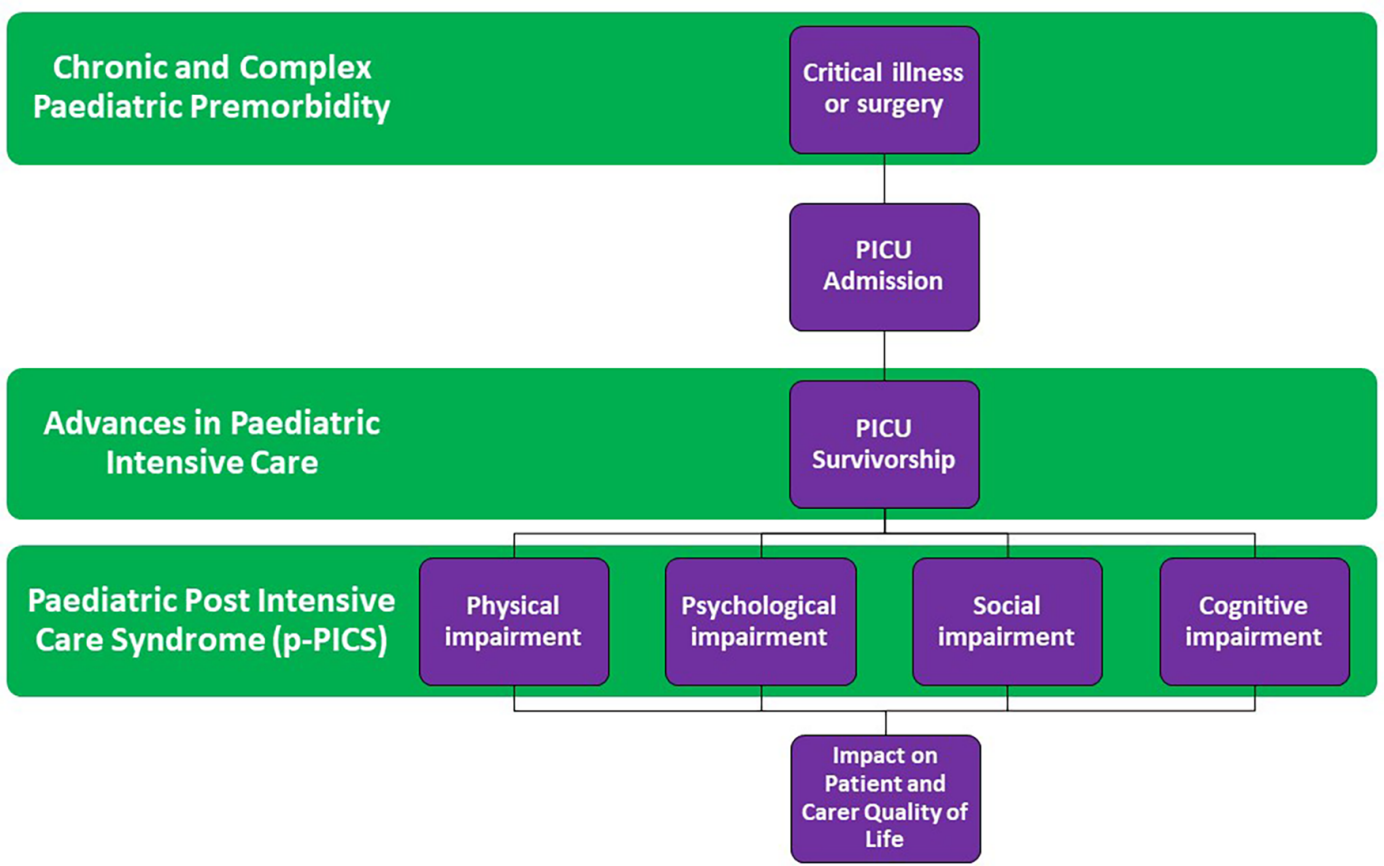
Conceptual model of pediatric post-intensive care syndrome.

The complexity of defining PICS in children is even more challenging given the timing of critical illness during childhood development in addition to the multitude of premorbid conditions that exist in the PICU population. An increasing number of patients admitted to PICU have chronic and complex medical backgrounds or known genetic disorders (8) which impact their developmental trajectory independent of post-PICU morbidity. Accepting these challenges, many authors have attempted to define PICS in paediatrics (PICS-p). PICS-p refers to children discharged from the PICU who subsequently develop new or additional long-term impairments in their physical, mental and/or, psychosocial health (9, 10). This may lead to difficulty in activities of daily living and inability to achieve normal or premorbid function and development, with subsequent impacts on quality of life and additional carer burden on families and caregivers (11, 12). The complex interplay of these paediatric specific factors is highlighted in a conceptual model in Figure 1.

Current gaps in our understanding of PICS-p are a major impediment to the development of a consistent approach to diagnosis and screening for this syndrome and to the future development of effective and robust treatment and mitigation strategies. The benefit of multidisciplinary early intervention for optimizing development and psychosocial health in paediatrics is well-established in other contexts and subspecialties, for example in the premature neonatal population and children at risk of cerebral palsy (13, 14) and it is logical that similar benefits could extend to the vulnerable cohort of paediatric intensive care survivors. Thus, further research is required in this important area of paediatric intensive care and a unified and prioritized approach is necessary to ensure that practical, achievable and applicable advances can be made.

## The “What”: what should be the focus of follow-up?

**Figure 2:**
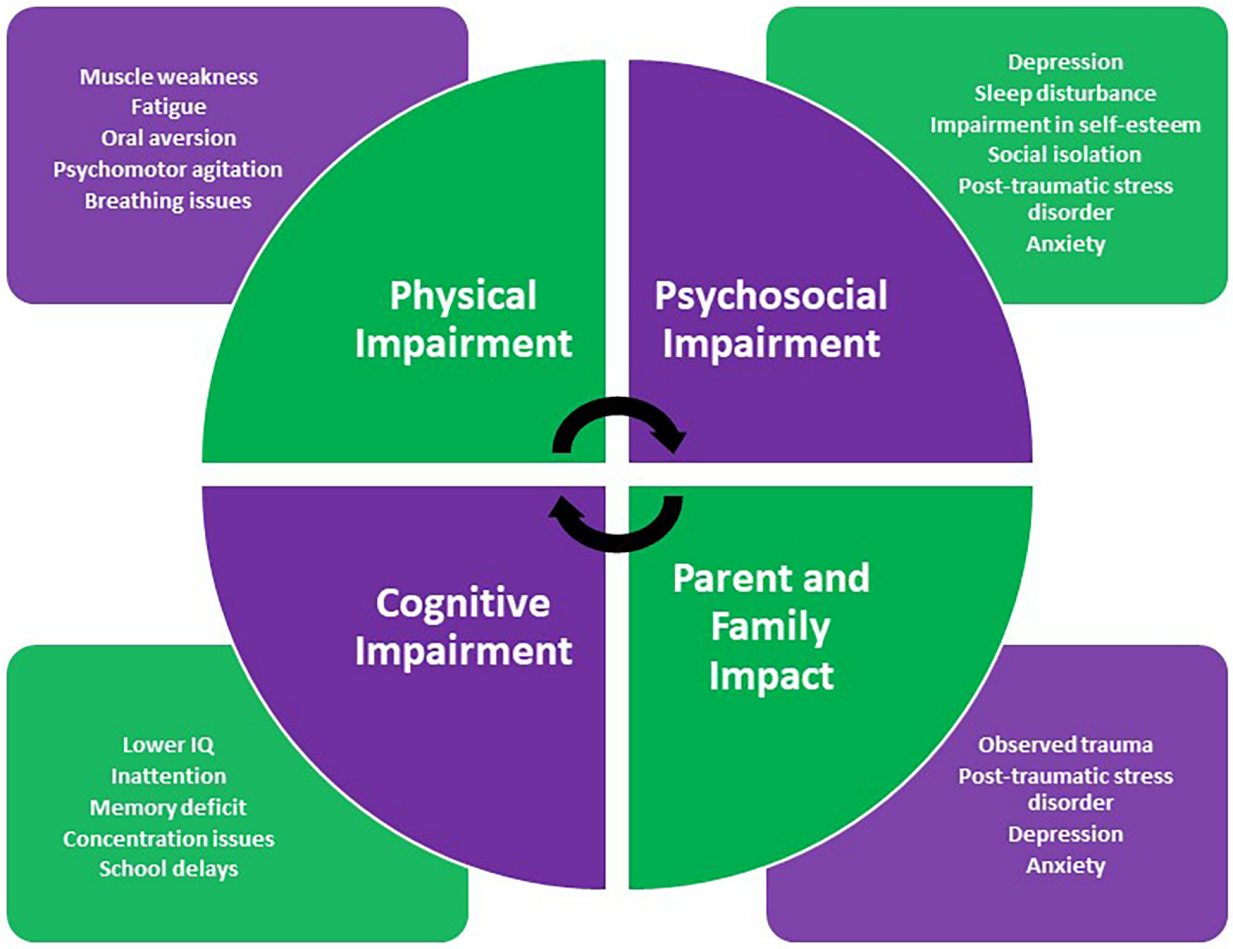
Characteristics of pediatric post-intensive care syndrome.

The main domains of PICS-p include physical, psychological, social and, cognitive impairment, all of which are associated with poorer quality of life for patients and carers (4). Here, we summarise the literature available regarding the impact of a PICU admission on these individual domains with their characteristics highlighted in Figure 2.

### Physical impairment

Impairment in physical functioning following PICU admission is well established in the literature (15). Risk factors contributing to physical impairment include patient-related factors such as respiratory and other musculoskeletal weakness, intervention-related risk factors such as requirement for ventilatory or circulatory support, prolonged periods of immobilization and, pharmacological risk factors including prolonged use of sedating medications. In younger children, impairment has been noted in both gross motor and fine motor function (16, 17).

In a scoping review, Ong et al. (2) noted rates of functional physical impairment following PICU admission as high as 36% at discharge and 13% at 2 years following discharge. They identified risk factors including severity of illness, presence of organ dysfunction, younger age and, length of ICU stay. Other studies note that while psychosocial impairment appears to be more prevalent in PICU survivors, physical impairment may be more severe and persistent (2, 18).

Assessing the frequency and impact of PICS-p is complicated by the lack of standardized instruments or tools used to assess physical impairment in children following PICU admission. Many are described in the literature, including health-related quality of life instruments, neuromotor development and functioning tools, global health functioning instruments and structured physical examination and, assessment by clinicians. In their systematic review, Bossen et al. (15) report that the Functional Status Scale (FSS), Child Health Questionnaire (CHQ) and Pediatric Quality of Life (PedsQL) inventory are the most frequently used instruments when assessing physical function following paediatric intensive care (15). Inconsistency in measurement creates difficulties in comparing studies and challenges in identifying effective strategies to recognize and address significant functional impairments.

### Psychosocial impairment

Historically, PICU long-term follow-up data has focused on mortality and the physical aspects of morbidity following discharge. More recently, increased focus on the psychological health and wellbeing of these children has revealed some sobering findings (19, 20) with an increased risk for psychiatric disorders and psychological and behavioral difficulties reported.

The literature shows that up to 25% of children demonstrate psychological and behavioural difficulties during the first year following PICU discharge (21). In their review, Rennick et al. (22) highlight a range of psychological symptoms in children post PICU discharge including increased anxiety, impairments in self-esteem and emotional wellbeing, sleep disturbances and, social isolation. Older school-aged children reported medical fears and anxieties, hallucinations and changes in their sense of self with significant impact on their social relationships and friendships (22). These findings are consistent with findings from Ducharme-Crevier et al.'s (16) follow-up cohort in which 21% of school-aged children were experiencing school delays and 20.5% had sleep disturbance within 2 month's post PICU discharge (16). Of note, much of the literature around psychological impairment following PICU discharge has focused on impairment within one year of discharge and has not assessed these outcomes in younger children whom often constitute a larger proportion of the PICU patient cohort. Future studies including the “Caring Intensively” study aim to address these gaps and their results are eagerly anticipated (21).

The literature shows high point prevalence of significant psychological illness within the first year following PICU discharge, similar to that of paediatric cancer survivors and children following traumatic injury (23). Of notable concern is the increased prevalence of post-traumatic stress disorder (PTSD) and major depressive disorder in PICU survivors with evidence to suggest the risk to be as high as 28% and 13% respectively (23). Other studies indicate that children who were more severely ill and had exposure to a higher number of invasive procedures in the PICU demonstrated an increased number of symptoms of PTSD (24, 25). Acute Stress Disorder has also been highlighted as being prevalent in this cohort (20, 26).

Like assessment of physical impairment following PICU discharge, no standardized tool or instrument has been recognised for the assessment of the psychological impact of PICU admission. Tools utilised variably in the literature include the PedsQOL inventory as well as the Impact of Events scale (23). Rennick's group have innovatively utilised some of their studies to guide the development of two child self-report measures of post PICU discharge psychological distress—the Children's Critical Illness Impact Scale and the Young Children's Critical Illness Impact Scale pictorial version (27, 28). In addition, Kazak et al. have proposed an integrative model of pediatric medical traumatic stress that has been used in the literature to further our understanding of diagnosing and treating PTSD in these patients (29).

### Cognitive impairment

Cognitive impairment following critical illness is well documented in the adult population with identified risk factors including delirium, prolonged use of opioids and sedatives, glucose dysregulation and, hypoxia (30, 31). Although of crucial importance to our understanding of PICS-p, long-term cognitive outcomes in critically ill children following PICU discharge are yet to be well characterised. A major limitation in understanding these outcomes is the impact of pre-existing cognitive deficit or delay that exists in many of the children that require paediatric intensive care. Few studies report the premorbid cognitive function of these children prior to PICU admission (8).

Amongst studies that do assess cognitive impairment in PICU survivors, intelligence, memory, and, attention have all been reported to be adversely affected. Much of the literature evaluating intelligence in PICU survivors has been in the sepsis patient cohort (32) and a number of studies report these survivors to have below average IQ when compared to healthy community controls following discharge from the PICU, with verbal IQ more predominantly affected (33, 34). Deficits in spatial working memory, sustained attention and, visual attention have also been reported (35, 36). A retrospective analysis by Bone et al. (37) found that a trauma diagnosis, unscheduled or non-elective admission to PICU, primary oncological diagnosis or, primary neurological diagnosis were independently associated with acquiring cognitive disability following PICU discharge. Interventional risk factors included invasive mechanical ventilation, renal replacement therapy, CPR and extracorporeal membrane oxygenation (37).

In contrast to other domains, assessment of cognitive dysfunction in paediatric patients is relatively robust and standardized, with the availability of a number of well accepted tools and instruments. Most studies in the literature employed one of the Weschler instruments to assess intelligence and either the Children's Memory Scale or, the Cambridge Neuropsychological Test Automated Battery to assess memory with the latter tool also being used to assess attention and executive function (32). Other studies have utilised the Pediatric Overall Performance Category scale and the Pediatric Cerebral Performance Category scale (38–40).

### Parent, caregiver and family impact

The significant impact on parents and caregivers following admission of their child to the PICU is well described in the literature. This includes both direct impacts of being in the PICU themselves whilst accompanying their children and indirect impacts of carer burden following the development of new or additional functional impairments associated with PICU survivorship (20, 41, 42). Some parents also suffer observed trauma resulting from events witnessed in the PICU unrelated to their own children (43).

As in children, symptoms of acute stress disorder and post-traumatic stress disorder are particularly prevalent in parents of PICU survivors on follow-up (44). Nelson et al. reported up to 84% of parents may have subclinical symptoms of PTSD following discharge from PICU with prevalence rates of diagnosed PTSD as high as 21% (45). Other symptoms include those of depression and anxiety (46, 47). Addressing these comorbidities in parents of PICU survivors is paramount as poor parental mental health and wellbeing reduces caregiver capacity, may lead to inability to work and, in addition, the development of chronic illness which will place additional stressors on the family (48). Furthermore, mental wellbeing of children has been associated with the mental wellbeing of their parents (46). Early identification of at risk parents with tools such as the Parental Stressor Scale and the Post-traumatic Adjustment Scale with subsequent provision of psychological support has already been shown to be beneficial and further research is warranted (49).

Furthermore, although the impact of paediatric illness on siblings has long been documented (50), the detrimental effects of PICU admission on siblings has only recently been considered. Siblings visiting the PICU have been shown to experience a range of physical, emotional and, social responses and thus must be included in future attempts when following up these vulnerable families (51, 52).

## The “Who”—who should be followed up?

Having established that the impacts of paediatric critical illness are significant, the need to provide appropriate follow-up thus becomes imperative. The key questions are then “who should be followed up” and “who should follow them up?”. It is important to note that although many subspecialty patients are followed up by their treating physicians, this may focus on specialised outcomes (for example surgical recovery), instead of those noted in PICS-p. In a world of finite resources, both human and financial, it may be challenging to provide comprehensive follow-up for every child admitted to intensive care. Equally, this is in many cases unnecessary with many patients transiting briefly through PICU without becoming “critically ill”. It is therefore important to consider those most likely to benefit from a follow-up service in terms of neurodevelopmental and quality of life outcomes.

There have been efforts internationally to follow-up PICU survivors in specific high-risk subgroups, of note in those children following extracorporeal membrane oxygenation (ECMO) support, complex congenital cardiac surgery (including in those with single ventricle physiology) and, cardiac arrest. Namachivayam et al. (53) retrospectively followed-up long-stay patients admitted to PICU for >28 days and found that more than two-thirds had unfavourable outcomes in functional status and quality of life (53). A retrospective cohort study of hematopoietic stem cell transplant (HSCT) patients following PICU admission demonstrated that survival with new functional morbidity was as prevalent as PICU mortality (54).

### ECMO patients

The Extracorporeal Life Support Organisation (ELSO) has published guidelines outlining follow-up recommendations for Neonatal and Paediatric ECMO patients extending from infancy to adolescence which the majority of centres internationally are not currently able to provide (55). Notably, the Netherlands offer a post-ECMO follow-up program for neonatal ECMO survivors in which lung function, growth and neurodevelopment are regularly assessed until 18 years of age. At 8-year follow-up, 79% of eligible children were assessed and found to have average intelligence with subtle cognitive problems in the areas of concentration and behaviour (56). In a single institution study in London, neonates and children who required ECMO for respiratory failure were followed up at one year post discharge with specific neurodevelopmental concerns identified in 30% of the cohort (57). Another institutional perspective from Australia found that 25% of patients undergoing ECMO support in early infancy had moderate to severe neurodevelopmental impairment, with gross motor and language the most affected developmental domains (58).

In the United States, a study from Boston Children's Hospital reported that children under 3 years who required ECMO for cardiac indications were found to have significant delays in language, motor and, adaptive functioning with risk factors for poorer outcomes including older age at first cannulation, male sex, complex cardiac disease and, longer length of hospital stay (59). A pilot study of children under five years of age who required ECMO at Texas Children's Hospital reported that 46% of patients assessed in clinic were diagnosed with developmental delay with a significant association noted between developmental delay on follow-up and post-ECMO MRI abnormalities raising the question about post-ECMO neuroimaging as a potential standard of care in this vulnerable cohort (60).

Of note, a quality improvement initiative in the United Kingdom attempted to establish a collaborative and standardised clinical assessment and management pathway for neurodevelopmental outcomes between the various ECMO centres however due to insufficient resources, the recommended assessments are not currently provided as standard of care (55).

### Congenital heart disease and complex therapies

Impact on neurodevelopmental outcomes following complex cardiac surgery is well established in the literature. In a study from the Sick Kid's Hospital in Toronto, neurocognitive outcomes of neonates undergoing arterial switch operation were assessed at 18 months using the Bayley Scales of Infant and Toddler development. Age at surgery and greater time with open chest were associated with lower language scores while length of stay was associated with lower cognitive scores (61). Adults in France who had had an arterial switch operation underwent IQ testing and assessment of health-related quality of life, with findings showing that cognitive morbidities commonly reported in children and adolescents with complex congenital heart disease persist into young adulthood in these individuals (62).

In Western Canada, a Complex Paediatric Therapies Registry exists for longitudinal follow-up of certain patients undergoing complex cardiac surgery, heart transplantation, ventricular assist device support, ECMO support, chronic renal dialysis and, following in-hospital cardiac arrest. Based on the neonatal follow-up model, the program includes multidisciplinary assessments with neurodevelopmental intervention, quality improvement and outcomes research. This program has achieved a 96% follow-up rate over two years (63).

### Cardiac arrest

Cardiac arrest survivors recruited to the THAPCA-IH trial who received ECPR, ECMO after ROSC and no mechanical circulatory support were assessed 12-months post arrest using an adaptive behaviour questionnaire, neuropsychological testing and, neurological examination. All children included had typical neurobehavioral function prior to cardiac arrest as assessed by caregiver questionnaire. Despite this, 60% of ECPR survivors under six years of age were found to have cognitive performance-based skills in the impaired or severely impaired range. Caregiver reported adaptive behaviour declined from pre-arrest baseline in all domains—particularly daily living and motor functioning. Overall, the ECPR survivors had similar outcomes to other in-hospital cardiac arrest survivors enrolled in the trial (64).

As noted from experiences of the groups explored above, high-risk events such as cardiac arrest, severity and number of organ failures and, PICU length of stay seem to correlate with significant long-term morbidity. Whilst focusing on high-risk groups is a reasonable starting point for outcomes research and follow-up, a longer-term goal should be to offer follow-up to the broader group of PICU survivors, including those that do not belong to these more researched groups and thus may be missed. For example, Shein et al. (65) evaluated neurodevelopmental outcomes of healthy children admitted to PICU with bronchiolitis using both questionnaires and in-person standardised assessments. Although those with known developmental delay at PICU admission were excluded, only 3 of 18 of the children performed within normal limits on assessment 1–2 years later (65).

## The “How”—concepts to consider

Given the disparity in research methodology in studies investigating PICS-p and long-term outcomes in paediatric intensive care, there is little literature available to guide longitudinal screening and follow-up in PICU survivors. Whilst the importance of follow-up in the PICU has been widely recognised by paediatric intensivists globally, there are no published follow-up clinic protocols or operating procedures. The few published prospective cohort studies of follow-up clinics that do exist are summarised in Table 1 below. A recent review into follow-up practices of all of the PICUs in the United Kingdom and Ireland revealed that only four units had post-discharge follow-up protocols (69). Another survey completed by Williams et al. (70) with respondents from 60 institutions within the United States, Canada, Australia and the United Kingdom noted only 17 active follow-up programs even though more than 80% of the 111 respondents highlighted the importance and direct benefits of such follow-up clinics (70).

**Table 1:** Current international studies evaluating long-term follow-up post pediatric intensive care.

Authors	Journal	Year	Country	Inclusion/exclusion criteria	Follow-up clinic details	Tools used	Main findings
De Sonnaville et al. (66)	Pediatric Critical Care Medicine	2023	Netherlands	All children aged 0–18 years of age who were admitted to the PICU were included. Patients who received similar follow-up elsewhere (endocrinology, palliative care, sleep team, rehabilitation) were excluded as well as patients who were admitted for elective post-operative care	3–6 months following PICU discharge by paediatric intensivist and multidisciplinary team including subspecialists. Planned follow-up of children < 6 years of age when they reach 6 years old	Strengths and Difficulties questionnaire PedsQL Children's Revised Impact of Event Scale	307 patients, median age 14 months 52.1% respiratory illnesses, 15.6% shock, 11.5% trauma Median LOS 4 days 53.1% required mechanical ventilation, 15% required inotropic agents Important outcomes and findings: – 18.9% of patients had sleep disturbance – 18.6% of patients who had neurocognitive testing (43/307) had a full scale intelligence quotient (FSIQ) less than −1 SD When compared to the general population, mothers of PICU survivors had higher rates of PTSD, anxiety, depression and distress The prevalence of clinical scores for behavioural and emotional functioning and health-related quality of life was higher in patients compared to the general population
Ducharme-Crevier et al. (16)	Pediatric Critical Care Medicine	2021	Canada	LOS > 4 days, mechanical ventilation ≥ 2 days, non-invasive ventilation ≥ 4 days Cardiac patients excluded	2 months following PICU discharge by paediatric intensivist using validated questionnaire	PedsQL ASQ Hospital anxiety and depression scale (HADS)	132 patients, mean age 44 months 40.9% respiratory illnesses, 7.6% head trauma, 7.6% septic shock Mean LOS 28.5 days 61% required mechanical ventilation Important outcomes and findings: – 21.4% of patients had school delay – 20.5% of patients had sleep disturbance – 15.9% of patients had breathing issues – 12.1% of patients had oral aversion – 9.9% of patients had voice changes – 9.8% of patients reported fatigue – 7.6% of patients had muscle weakness – Longer PICU stay and presence of premorbid conditions led to lower PedsQL scores however pre-PICU QOL not assessed – 42% of parents had signs of anxiety – 29% of parents had signs of depression
Knoester et al. (67)	Intensive Care Medicine	2008	Netherlands	Previously healthy children admitted to PICU with an acute life-threatening illness who were admitted for >7 days Patients with underlying illnesses or those being admitted for elective surgery or following abuse or self-intoxication were excluded. Families unable to complete the Dutch questionnaire were also excluded.	3 months following PICU discharge by paediatric intensivists using structured history taking	Pediatric cerebral performance category (PCPC) Pediatric overall performance category (POPC)	186 patients, mean age 1.4 years 41% respiratory illnesses, 24% trauma, 17% shock Median LOS 6 days 81% required ventilatory support (unclear I + V vs. NIV) 25% required vasoactives Important outcomes and findings: – 15% of patients had behavioural problems – 13% of patients had delayed psychomotor problems – 9% of patients reported concentration issues – 9% of patients had sleeping issues – 9% of patients had eating problems – 22% of school aged children had problems at school Three months post discharge only 31% of children were healthy, 30% had previously unknown underlying illness, 39% had acquired morbidity
Gledhill et al. (68)	Advances in Critical Care	2014	United Kingdom	Children aged over 8 years Children with underlying neurological disease were excluded Families unable to speak English were excluded.	6 weeks following PICU discharge by paediatric intensivist and paediatric psychiatrist	Strengths and Difficulties questionnaire General Health Questionnaire Impact of Events Scale	4 out of 14 families attended follow-up Mean age 11.8 years Mean LOS 6.5 days All admissions were for respiratory issues No children had major physical problems at follow-up All 4 reported psychological difficulties: – 3 had sleep issues – 2 had anxiety – 2 had PTSD All 4 mothers attending reported psychological difficulty: – 2 had PTSD

Of note however, international collaboration has led to the establishment of a multi-national pediatric critical care core outcome set which characterizes PICU survivorship and has provided an essential tool in standardizing future research in this crucial area (71–73).

**Figure 3:**
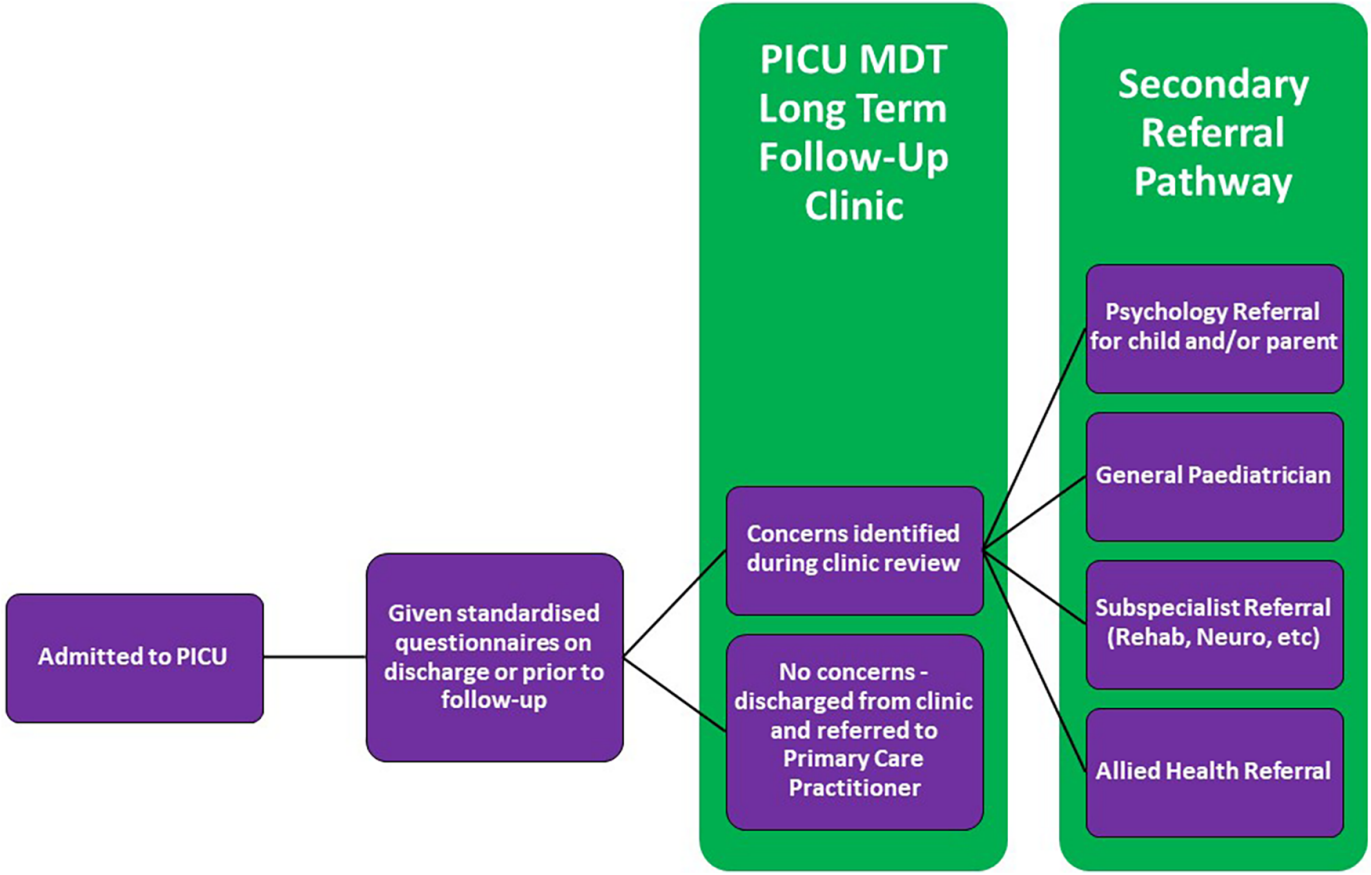
Conceptual model of a pediatric intensive care follow-up clinic.

Although further research is required to establish the best clinical pathway for follow-up in the PICU, we propose some basic principles inferred from the current literature. A conceptual model for a PICU follow-up clinic is described in Figure 3.

### A multidisciplinary approach

As established in this review, identification of PICS-p requires a multidisciplinary approach and thus a PICU follow-up program would benefit from being modeled on the well-established multidisciplinary team follow-up provided by neonatology developmental follow-up clinics. As evidenced in the studies above, such a clinic could be led by an intensivist although the adult critical care experience has also shown the benefit of nurse-led follow-up programs (74). The benefits of an intensive care trained clinician leading the clinic are many including that of being able to provide contextual experience of critical illness and intensive care therapies.

In Williams et al.'s (70) survey of PICU follow-up programs, approximately one third of the institutions involved noted physician only programs despite the strong consensus between respondents about the need to assess post PICU discharge morbidity across multiple domains (70). The need for a multidisciplinary approach to follow-up in the PICU has been routinely discussed in the literature as explored in this review; the importance of which is well established in the adult critical care literature both pre- and especially post- COVID-19 (75, 76). Given the known deficits that tend to occur in these children, the multidisciplinary team should have a multi-domain focus incorporating physiotherapy, occupational therapy, neuropsychology and, social work with consideration of liaisons to educational institutions and other government and, disability support agencies. Thus based on this evidence, a PICU follow-up clinic should be led by an experienced paediatric clinician with relevant expertise in post-intensive care issues and morbidity overseeing a wider multidisciplinary team comprising of the allied health clinicians as highlighted above.

### Addressing current gaps

An argument can be made that many of the complex patients that are admitted to PICUs have primary teams that will follow them up after their discharge. However, for the appropriate patient subset, a PICU specific follow-up clinic offers potential benefits of additional focus on rehabilitation and psychological wellbeing that may not be considered in some subspecialty or more disease centric clinics. The risk of assuming that these children always have robust follow-up is not insignificant. As shown in their prospective follow-up study, more than one third of Ducharme-Crevier et al.'s (16) cohort did not have a primary physician or treating team to follow-up with (16). The children particularly at risk are those that are previously well who require a short but often significant admission to PICU—for example the well infants who are admitted for mechanical ventilation for bronchiolitis or vasopressors for sepsis. These children often may not have a paediatrician and may get lost to follow-up.

Further research is required to identify these gaps to guide efficient PICU follow-up and prevent misuse and duplication of established services and infrastructure. Given that the purpose of a PICU follow-up program should be to screen for red flags in known areas of potential impairment in children post PICU discharge, follow-up beyond the initial multidisciplinary review should be in collaboration with other specialists in order to utilise their expertise and existing services to ensure that best care is provided (77). With further research into long-term follow-up in the PICU, cost benefit analyses must be considered to evaluate if such initiatives are sustainable and provide evidenced benefit.

### Including the right patients at the right time

Given the highlighted heterogeneity in the PICU patient population, an initial approach to follow-up may be to focus on higher risk groups that undergo significant and invasive therapy including ECMO support, complex cardiac and other surgery, prolonged or multiple organ failure and, following cardiac arrest. Although the exact timeframe for when this follow-up should occur is not yet apparent, further investigation into PICS-p and further delineation of its “diagnostic criteria” may lead to the identification of relevant factors that may warrant an earlier review with the eventual aim of developing inclusion and exclusion criteria for follow-up.

What is evident in the literature, however, is that follow-up in these vulnerable children is required both in the acute phase but also in the longer term as many deficits may not be apparent until much later in life including at times into young adulthood (78–80). This well documented “growing into deficit” concept that suggests that some deficits may present later in life needs to also be carefully considered when establishing follow-up programs, particularly when deciding on follow-up duration and timeframes (79, 81).

## Conclusion

As mortality rates for children with critical illness continue to improve, increasing PICU survivorship is resulting in significant long-term consequences of intensive care in many vulnerable patients. Impairments in physical, psychosocial and cognitive function have been well documented and although the importance of establishing follow-up programs is acknowledged, there remains a significant amount of work and further research to be done to address gaps in our understanding of how to establish effective and sustainable follow-up programs that allow these children to live their best and most fulfilled lives beyond the PICU. With this in mind, we propose the following research priorities that should be addressed in the literature going forward:

1. Further definition of PICS-p as an entity with development of standardized diagnostic criteria
2. Selection and validation of standardized tools to assess the different types of impairment that children may experience following PICU admission
3. Development of consensus guidelines on optimal processes and priorities for PICU follow-up services that can be practically adapted and implemented to institutions worldwide
4. Ongoing evaluation and research into the benefits of early recognition and intervention on the various morbidities that exist in PICU survivors
